# Correspondence between Parents’ and Adolescents’ Sleep Duration

**DOI:** 10.3390/ijerph19031034

**Published:** 2022-01-18

**Authors:** Eunyoung Jeon, Nayoung Kim

**Affiliations:** 1 Department of Nursing, Daegu University, Daegu 42400, Korea; jeoney@daegu.ac.kr; 2 Department of Nursing, Daegu Haany University, Gyeongsan-si 38610, Korea

**Keywords:** parents, adolescents, sleep duration

## Abstract

This study explored the correspondence between adolescents’ sleep duration and that of their parents and identified the factors affecting the appropriate sleep duration for adolescents. The data of 795 adolescents from the Korea National Health and Nutrition Examination Survey (2015–2018) were analyzed. We used Cohen’s kappa coefficient to measure the correspondence between adolescents’ sleep duration and that of their parents. A multiple logistic regression analysis was used to identify the factors affecting adequate sleep duration among adolescents. Our study found that factors such as adolescents’ gender, father’s education level, and drinking among adolescents and parents influenced the adolescents’ sleep duration. Second, a higher correspondence between the sleep duration of adolescents and that of mothers (Kappa = 0.213, *p* < 0.001) was found compared to that of fathers (Kappa = 0.064, *p* = 0.031). Finally, an adequate sleep duration among adolescents’ mothers was a major factor that influenced the adequate sleep duration of adolescents (OR = 2.494, 95% CI = 1.850–3.362, *p* < 0.001). Therefore, when organizing adolescent sleep education and management programs in various community sleep management institutions, the main caregiver’s sleep duration management and family drinking management should be combined. Additionally, gender equality awareness should be improved for parenting, including monitoring adolescents’ sleep accordingly.

## 1. Introduction

Adolescence is a critical period of rapid transformation and development. During this period, lifestyle choices can affect adolescent health and possibly impact future generations, when young people start families of their own [1]. Among lifestyle habits of adolescents, sleep plays an essential role in maintaining a healthy life, promoting normal growth, emotional stability, and cognitive function [2,3]. According to the Organization for Economic Co-operation and Development’s (OECD) 2015 announcement, [4] the average sleep duration of Koreans ranked the lowest among the 18 countries surveyed. In another study comparing sleep duration featuring nine countries including the UK, Germany, Italy, and Denmark, sleep duration in Korean adolescents was significantly lower than in other countries [5]. Additionally, it was revealed that life satisfaction in Korean children and adolescents is the lowest among OECD countries [6]. Based on a 10-point scale, the average life satisfaction of children and adolescents in Korea was 6.6 points, while the average in the other 27 countries was 7.6 points [6].

The National Sleep Foundation (NSF) suggests that the appropriate sleep duration for teenagers (14–17 years old) is 8–10 h and 7–9 h for adults (18–25 years old) [7]. However, the average sleep duration of middle and high school students in Korea is 7.3 h; 7.4 h for middle school students (14–16 years old), and 6.0 h for high school students (17–19 years old) [8]. Asian adolescents tend to sleep later than teenagers in North America and Europe, their total sleep duration shorter, and rate of daytime sleepiness higher [9]. In Korean adolescents, issues such as mounting pressure to achieve academic excellence and ace college entrance exams, as well as rampant addiction to smartphones highly impact the ability to attain adequate sleep [6].

Meanwhile, chronic insufficient sleep can negatively affect adolescent health. In addition to research that points out the various physiological and cognitive consequences of poor sleep, several studies have reported that short sleep durations lower life satisfaction and increase the risk of suicidal ideation [3,10,11,12]. A 2020 youth statistics survey noted that suicide was the number one cause of death for Korean adolescents that year [8]. Substance use is also connected [13] as well as increased risky behavior and risk of accidents and injuries [14]. Chronic sleep deprivation in adolescents is accompanied by excessive sleepiness during the day, causing poor academic achievement [15] and negatively affecting school life adaptation [12]. In addition, changes in hormone secretion in the body due to lack of sleep can affect appetite and energy metabolism, increasing the risk of obesity [12,16,17].

Sleeping habits are influenced by family environments, particularly by parents [18]. The quality of the parent-child relationship is connected to better sleep [19]. Parent-set bedtimes can positively affect an adolescent’s sleep behaviors [20]. In a youth study in South Australia, adolescents with set bedtimes had earlier bedtimes, attained more sleep, and exhibited improved daytime wakefulness and less fatigue [20]. In a study on risk factors associated with sleep deprivation among school-aged children in China, the shorter sleep duration of parents influenced the sleep duration of the children [21]. Family context plays an important role in adolescents’ sleep duration. A cohesive environment can provide a sense of stability and security that is necessary for their adequate sleep [22]. However, if the environment is stressful, for example in a family belonging to lower socioeconomic (SES) status, the stress affects the adolescents and results in higher intra-individual variations among their sleep durations [18]. Based on previous studies, it can be seen that parents’ supervision can affect their adolescent’s sleep duration.

Existing studies on adolescents’ sleep patterns in Korea have primarily been conducted with a focus on mental health, emotion, and health risk behaviors [11,17]. In foreign countries, parental monitoring of adolescents’ sleep duration has been conducted recently [20], as well as investigations about the relationship between parenting attitudes and sleep [19]. Certain studies related to family contexts have been conducted as well [18]; however, few studies have examined the agreement or correspondence of sleep patterns in adolescents and their parents. To fill this gap, this study aims to provide preliminary data to develop parent-adolescent education programs by examining the degree of correspondence of sleep duration of adolescent participants to that of their parents. It also aims to identify the factors affecting the appropriate sleep duration of adolescents. 

First, we identify the general characteristics of adolescents and parents. Second, we identify the sleep duration of adolescents according to these general characteristics. Third, we identify the degree of correspondence between adolescents’ and parents’ sleep durations. Finally, we identify the factors affecting the appropriate sleep duration of adolescents.

## 2. Materials and Methods

### 2.1. Study Design

**Figure 1:**
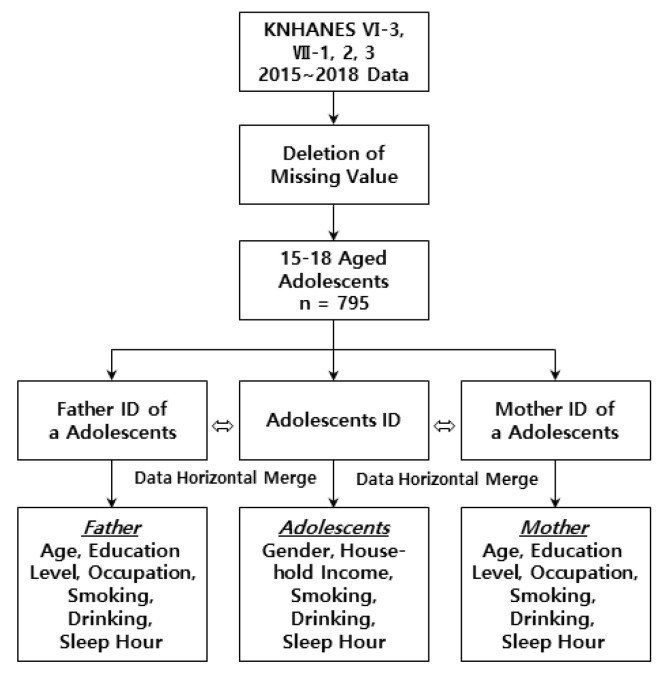
Study model design.

We conducted a descriptive exploration study with secondary analyses of raw data from the years 2015, 2016, 2017, and 2018 of the Korea National Health and Nutrition Examination Survey (KNHANES) conducted by the Korea Disease Control and Prevention Agency (KDCA). KNHANES is an ongoing surveillance system in South Korea that assesses the health and nutritional status of Koreans, monitors trends in health risk factors and the prevalence of major chronic diseases and provides data for the development and evaluation of health policies and programs in Korea [23]. The survey consists of a health examination, health interview, and nutrition survey, using a health interview questionnaire. Our analyses seek to examine and define the degree of correspondence between parents’ and their adolescent’s sleep durations and the factors affecting the adolescent’s sleep duration (Figure 1). In this study, the adolescent questionnaire includes gender, the house-hold income of the parents, drinking, smoking, and sleep duration, while the parental questionnaire includes age, education level, occupation, drinking, smoking, and sleep duration questions. Several studies were cited during the selection of variables. Namely, a study based on the Korea Youth Risk Behavior Survey, on predicting factors influencing the sleep duration of adolescents in Korea identified factors such as gender, parents’ educational level, smoking, drinking, and economic status [24]. Based on the data from the Korean Children and Youth Panel Survey, parents’ occupation was identified as a factor predicting the poverty of children’s sleep duration and sleep time [25]. In addition, gender [26,27], parental drinking [28,29,30], and parental sleep duration [21,31] were found to have an effect on adolescents and children’s sleep duration, these were selected as a variable that can be used in the KNHANES data.

### 2.2. Participants 

Raw data from the years 2015, 2016, 2017, and 2018 of the KNHANES were analyzed for this study. The participants of this study included adolescents from two-parent households aged 15 and 18 years. The connection data were formed by finding and matching the adolescent ID with their parents’ IDs. The number of parent and adolescent IDs collected was 159 in 2015, 176 in 2016, 167 in 2017, and 293 in 2018—a total of 795.

### 2.3. Measures 

Sleep duration was used as a dependent variable to determine the degree of agreement of sleep duration patterns between adolescents and parents. To determine the degree of agreement or correspondence through Kappa statistics, the sleep duration of adolescents, fathers, and mothers was classified into three groups: less than 6 h, 7 to 8 h, and 9 h or more. Sleep duration was classified based on the ALAMEDA 7 [32], which is a common tool applicable to adults and adolescents based on the appropriate standard of sleep duration of 7 to 8 h.

Demographic information included gender (male and female adolescents), parents’ household income, parental age, parents’ educational level, occupation, and whether the subjects smoked and consumed alcohol. Household income was classified into four groups: upper, middle-high, middle-low, and lower. It was classified according to the quartile of income by the number of monthly income-earning household members, calculated with the monthly average household equalized income. Parental age was classified as either under 50 or over 50 years of age based on the median value. Educational level was divided into below-high school graduation and college graduation or higher. Occupations were classified into non-physical workers such as managers and experts, office workers, sales and service workers, skilled agriculture professionals; physical workers in forestry or fishery work, skilled workers, simple labor workers; and inoccupation such as housewives and students. Smoking was classified into smokers (those who have smoked more than five packs (100 cigarettes) in their life and currently smoke) and non-smokers (those who have quit smoking in the past or have never smoked). Finally, drinking was classified into drinkers (those who drank more than once a month in the past year) and non-drinkers (those who had not had alcohol in their lifetime or drank less than one glass a month over the past year) [33].

The KNHANES data used in this study were approved by the Research Ethics Review Committee of the KDCA. Individual information from the participants studied could not be identified according to the procedure guidelines on the KNHANES website. This study was approved by the Institutional Bioethics Review Committee of D University (IRB No. 1040621-202101-HR-022).

### 2.4. Statistical Analysis 

We used open-source software R 3.6.2 (https://cran.r-project.org/bin/windows/base/old/3.6.2/) (accessed on 14 December 2021) for the following statistical analyses:

(1) A frequency test to grasp the general characteristics of the adolescents and parents.
(2) A cross-tab analysis (Chi-square test) to grasp the sleep duration according to the general characteristics of the adolescents and parents.
(3) A cross-tab analysis (Chi-square test & Cohen’s kappa agreement coefficient) to grasp the sleep duration of the adolescents according to the sleep duration of the parents.
(4) A multiple logistic regression analysis to grasp the factors affecting sleep duration (7–8 h) of the adolescents.

## 3. Results

### 3.1. General Characteristics of Adolescents and Parents 

Observing the general characteristics of adolescents displayed in Table 1, males accounted for 52.3% and females accounted for 47.7% of the adolescent participants. Household income was 38% in the upper tier, 33% in the middle-high tier, 22.8% in the middle-low tier, and 6.2% in the lower tier. The non-drinkers were 56.4% and 43.6% were the drinkers. The non-smokers were 94.3% and 5.7% were the smokers. As for sleep duration, 7–8 h was the most common duration at 49.9%, while 40.6% claimed to sleep fewer than 6 h, and 9.5% slept more than 9 h each night. 

According to the general characteristics of the parents displayed in Table 2, 75.3% of the fathers were under the age of 50, and 24.7% were above 50. 88.9% of the mothers were under the age of 50, and 11.1% above 50. 

As for the educational level of the fathers, 68.1% were high school graduates or below, while 31.9% were college graduates or above. For the education level of mothers, 72.6% were high school graduates or below, while 27.4% were college graduates or above. Jobs among fathers consisted of 60.1% non-physical jobs, 34.2% physical jobs, and 5.7% inoccupation. The mothers’ jobs included 57% non-physical jobs, 29.8% inoccupation, and 13.2% physical jobs. 

Drinking among the fathers was 35.6%, while 64.4% were non-drinkers. The fathers who smoked constituted 40.6% and 59.4% were non-smokers. Drinking among mothers was at 35.7%, while 64.3% were non-drinkers. The mothers who smoked constituted 3.8% and 96.2% were non-smokers. The fathers’ sleep durations were the most common, with 52.7% sleeping for 7–8 h, 43.0% less than 6 h, and 4.3% greater than 9 h. The mothers’ sleep durations were also the most common with 53.1% sleeping for 7–8 h, 44.2% less than 6 h, and 2.7% more than 9 h.

### 3.2. Sleep Duration according to the General Characteristics of Adolescents and Parents 

As seen in Table 3, the general characteristics of the adolescents and parents exhibited significant differences in adolescents’ sleep durations according to their gender, drinking frequency, the father’s educational level, the father’s drinking frequency, and the mother’s drinking frequency.

Depending on the gender, 38% of male adolescents slept for less than 6 h while 12.2% slept for more than 9 h; 43.5% of female adolescents slept less than 6 h, with 6.4% sleeping more than 9 h. Therefore, compared to female adolescents, a lower number of males slept less than 6 h, while a higher number of males slept more than 9 h. These indicate a significant difference (*p* = 0.012) in sleep durations based on gender. 

For adolescent drinkers, 45.2% slept for less than 6 h, and 5.5% slept for more than 9 h. At 37.1%, non-drinkers as well as 12.5% drinkers slept for greater than 9 h. An increased number of adolescent drinkers slept less than 6 h. Fewer participants who drank slept for more than 9 h, compared to the adolescent non-drinkers, showing significant difference (*p* = 0.001).

The percentage of teenagers whose fathers graduated college or higher was 55.9% and 4.7% for 7–8 h and more than 9 h of sleeping duration, respectively.

In the case of fathers graduating from high school or below, 47.1% slept for 7 to 8 h, and 11.6% slept for more than 9 h. The higher the father’s educational level, the more frequent 7–8 h of sleep adolescents obtained, and few had over 9 h of sleep, which was a significant difference (*p* = 0.003).

If the father was a drinker, 45.9% of adolescents obtained 7–8 h of sleep and 14.1% slept for more than 9 h. If the father did not drink, 52.1% obtained 7–8 h of sleep, and 6.8% slept more than 9 h. If the father did not drink, adolescents who slept 7–8 h had higher frequency, and fewer slept more than 9 h, indicating a significant difference (*p* = 0.003). 

If the mother drank frequently, 45.8% of adolescents slept for 7–8 h and 15.8% for more than 9 h. If the mother did not drink, 52.3% of adolescents obtained 7–8 h of sleep, and 5.9% slept for more than 9 h. When the mother was a non-drinker, similar to non-drinker fathers, a higher number of adolescents slept 7–8 h and few slept more than 9 h, indicating a significant difference (*p* < 0.001).

There was no significant difference in adolescents’ sleep durations depending on household income, adolescents’ smoking patterns, the age of the father, the age of the mother, the mother’s education level, the mother’s job, and the mother’s smoking patterns.

### 3.3. Correspondence between Parents’ and Adolescents’ Sleep Duration 

As a result of the Cohen’s Kappa coefficient shown in Table 4, the correspondence or agreement of the adolescents’ sleep duration with the father’s sleep duration was a slight agreement (Kappa = 0.064, *p* = 0.031), while the correspondence with the mother’s sleep duration was a fair agreement (Kappa = 0.213, *p* < 0.001).

### 3.4. Factors Affecting a 7–8 Hour Sleep Duration 

Through a multiple logistic regression analysis, the factors affecting adolescents’ ability to get 7–8 h of sleep were examined and exhibited in Table 5. All variables were input for analysis while the variables were controlled with each other. 

Results indicate that the mother’s sleep duration significantly influenced that of the adolescents. The factor affecting the adolescent’s 7–8 h sleep duration is the mother’s 7–8 h sleep duration, showing the odds ratio (OR) value of 2.494 (OR = 2.494, 95% CI = 1.850–3.362, *p* < 0.001). If a mother sleeps for 7–8 h rather than less than 6 h and more than 9 h, adolescent children are also highly probable to sleep for 7–8 h. 

## 4. Discussion

According to the results of this study, variables such as the adolescent’s gender, father’s education level, and drinking among adolescents and parents affected the adolescent’s sleep duration. First, in terms of gender, the sleep duration of female adolescents was longer than that of male adolescents in this study. This is consistent with the results reported in previous studies [26,27]. The results of this study show that the likelihood of an adolescent receiving adequate sleep of 7–8 h increased when the father had a higher educational level. A study by Lee (2016) reported that the higher a father’s educational level, the less time his adolescent probably spends playing games or watching TV, and instead engages in high performance yielding learning and leisure activities [34]. In a study by Song (2011), child-rearing time tended to increase when a parent’s level of education was higher [35]. This suggests that the time and efforts invested in raising children in a healthy and structured environment increase when the father has a higher level of education.

In this study, drinking was found to affect the sleep duration of adolescents. A study by Fucito et al. (2019), which featured college students who drank heavily, showed that bedtime and waking times were delayed on days following heavy drinking [36]. Further, the subjects who drank more reported shorter sleep durations on average [36]. In a study by Van Reen et al. (2016), it was found that bedtimes and waking times were highly delayed in adolescents and young adults who drank excessively compared to peers who drank less [37]. A study reported that college students who drank heavily and had poor sleep quality experienced more alcohol-related clinical symptoms than those who drank heavily and had high sleep quality [38]. Additionally, several studies have shown that various sleep problems during early adolescence are related to the early onset of drinking habits and that the risk of excessive drinking increases during late adolescence [39,40,41].

Furthermore, this study found that parental alcohol consumption influences adolescents’ sleep duration. A study by Kelly and El-Sheikh (2016) showed an association between increased parental drinking problems and shorter sleep duration in children, which was most evident in black children and children belonging to lower socioeconomic status [28]. A later study by Kelly and El-Sheikh (2019) longitudinally investigated the relationship between parental drinking problems and adolescents’ sleep. It showed adolescents’ sleep duration and efficiency decreasing gradually, over a period of time, if the father had a drinking problem, along with regular occurrences of adolescents requiring greater amount of time to wake up [29]. Another study reported that school-age children with alcoholic parents had shorter sleep durations and greater nighttime activities than children of parents with low alcohol consumption [42]. Hairston et al. (2016) also supported this study by suggesting that children of parents with high alcohol dependence had shorter sleep duration [30]. 

Several studies have shown that parental and adolescent drinking patterns are related to adolescents’ sleep problems, supporting the results of this study. To promote healthy sleep management of adolescents, parents must be educated on drinking habits along with relevant education for adolescents. 

This study revealed a higher correspondence between the mother’s and the adolescent’s sleep duration than between the father’s and the adolescent’s sleep duration. The results of the multiple logistic regression analysis revealed that adolescents whose mothers attained an adequate amount of sleep were 2.494 times more likely to get an adequate amount of sleep than those whose mothers did not. 

These results may reflect the cultural characteristics of child-rearing in Korean society, where traditional gender roles exist, with fathers perceived as the breadwinners and mothers as the caregivers. Therefore, mothers tend to devote a greater amount of time to child-raising compared to fathers. However, as the employment rate of married women in Korean society has increased, gender equality values have disseminated with fathers’ participation in child-rearing increasing in recent times. However, statistical data still shows a lack of father’s participation [4,43]. According to the 2019 time-use survey, Korean men spent 48 min on housework during the weekdays, an increase of nine minutes from the previous five years [43]. In contrast, the time spent by Korean women toward housework is 3 h and 10 min, a 12-min decrease from the previous five years, yet still four times that of men [43,44]. According to the OECD Report, the average Korean father spends six minutes a day with their children which is highly less than the OECD average of 47 min, being the lowest among OECD countries [44]. Despite the increase in the number of working women, mothers’ sleep duration is a powerful factor that influences adolescents’ sleep duration, indicating their greater influence on the adolescents’ sleep as compared to fathers. 

In a study that investigated the way in which adolescents’ bedtime affects the parents’ bedtime, focusing on the gender gap in Korean dual-income families, the effect of adolescents’ bedtime was stronger on the mother’s bedtime than the father’ s [45]. Even in circumstances in which both parents had to work the next morning, mothers reported being unable to sleep until later than the fathers [45], thus supporting the results of this study. Additionally, the previous study also revealed that sleep deprivation due to child-rearing was highly evident in mothers, and they experienced the burden of housework and child-rearing along with inequality in health [45]. Other studies have also reported the way in which women tend to suffer with a greater number of sleep disorders and shorter sleep durations than men [46,47] thereby supporting the results of this study. 

In China, a country that shows a similar degree of enthusiasm for education as Korea, a study revealed that parental sleep habits influenced children’s short sleep durations and emphasized the potential control these habits have on their children [21]. However, in this Chinese study, the OR of fathers with a sleep duration of less than 6 h was higher than that of mothers, and the OR for mothers with a sleep duration of 6–8 h was higher than that of fathers, without a statistically significant difference, which is different from the results of this study. Further, a study on Chinese adolescents also included fathers’ short sleep durations as a factor that influenced adolescents’ short sleep durations [31]. In the case of China, the OECD-reported average daily domestic work time among men as 91 min, which was lower than the OECD average of 139 min [44]. However, this was twice that of Korean men [44]. This difference is attributed to cultural differences despite similar contexts. 

Based on these studies, it is necessary for future research to closely examine the influence of the sleep duration of fathers and mothers on the sleep duration of adolescents through a comparative study of data among different countries. In addition, for the sake of children’s healthy development, it is necessary to raise awareness on gender equality for higher participation of fathers in childcare including sleep monitoring. 

Adolescent families face various difficulties. The most important parenting topics for adolescent parents were communication and conflict management, while health-based topics were related to sex, mental health, alcohol, and drugs [48]. In reality, it is particularly difficult to educate adolescents on the parent’s perspective since Korean adolescents may experience psychological difficulties such as stress and depression due to academic, career, and peer relationship issues, as well as other factors such as college applications, excessive competition, online games, and smartphone use. All of these factors contribute to poor sleep behaviors and shorter sleep durations.

Considering the aforementioned circumstances of Korean adolescents, sleep education for adolescents is crucial because sleep can affect degrees of life satisfaction and health. Since parent-set and supervised bedtimes can have a positive effect on sleep duration in adolescents [20], it is recommended that parents involve themselves more in their adolescents’ sleep behaviors and monitor them, so their children can adopt healthy lifestyles. The study by Rojo-Wissar et al. (2020) noted the relationship between mother-child bonding during childhood and the child’s sleep characteristics later in adulthood. They showed that higher levels of maternal care in childhood are associated with longer sleep duration, suggesting the necessity of forming a healthy attachment and bond with the mother before adolescence. Parental sleep supervision, based on healthy attachment and bonding with children from early stages of development, can allow the management of adolescents’ sleep behaviors that can lead to emotional stability and security. Since the adequate sleep duration of parents influences that of adolescents, awareness promotion and education programs for parents to lead a healthy life are also suggested. 

Lack of sleep among adolescents in Korea is an important issue. As national data was consulted for this study, moreover, to our knowledge, this study provides a novel contribution by associating adolescent data with that of their parents, which has rarely been conducted in this context. The clinical implications of this study influence parental education which, in turn, is essential in community clinics and educational institutions that can impact adolescent sleep duration. Thus, adequate sleeping patterns among both adults and adolescents should be included in educational content. In addition, the prevention of adolescent drinking as well as the management of parents’ drinking should also be included into educational content. This suggests that adolescent-related factors should not be considered solely when developing a sleep management program for adolescents due to the influence of their parents’ sleeping patterns. 

The limitations of this study resulted from selecting variables using secondary data. Consequently, it was not possible to include research variables on bedtime, time spent with parents and children, bedtime rules, genetic factors, as well as sleep quality. In addition, since this study was conducted in Korea, it may be difficult to generalize the results due to cultural specificity.

## 5. Conclusions

This study aimed to provide preliminary data for the development of health and sleep education programs for parents and adolescents by examining the degree of correspondence between the sleep duration of both. It also aimed to identify the factors that affect adequate sleep durations for adolescents. Study results reveal that, first, the factors that influence the sleep duration of adolescents according to the general characteristics of adolescents and parents were adolescents’ gender, their father’s education level, and drinking among adolescents and parents. Second, our study reveals a high correspondence between the sleep duration of adolescents and that of mothers compared to that of fathers. Finally, the adequate sleep duration of mothers is a major factor that influences the adequate sleep duration of adolescents. The results of this study suggest that when organizing adolescent sleep education and management programs in various community sleep management institutions, sleep duration management of the main caregiver and drinking management of the family should be combined. For the health of their children, fathers should aim to participate more actively in parenting, including sleep monitoring, to raise awareness of and address gender inequality. Since sleep is such a critical factor for adolescents in their development, it is necessary to educate parents about the importance of sleep duration management for the health and well-being of their adolescent children. 

Implications for future studies include a comparative study between countries on the sleep duration of parents and adolescents and a study on the development and effectiveness of adolescents’ sleep duration management programs for parents.

## Figures and Tables

**Table 1:** General characteristics of adolescents.

Variable	Frequency	Percentage
Gender		
Male	416	52.3
Female	379	47.7
House-hold Income		
High	302	38.0
Middle-high	263	33.0
Middle-low	181	22.8
Low	49	6.2
Drinking		
Drinker	347	43.6
Non-drinker	448	56.4
Smoking		
Smoker	45	5.7
Non-smoker	750	94.3
Sleep duration		
≤6 h	323	40.6
7–8 h	397	49.9
≥9 h	75	9.5
Total	795	100.0

**Table 2:** General characteristics of parents.

Variable	Father		Mother	
	Frequency	Percentage	Frequency	Percentage
Age				
≥50 yr	196	24.7	88	11.1
<50 yr	599	75.3	707	88.9
Education level				
≥College	254	31.9	218	27.4
≤High school	541	68.1	577	72.6
Occupation				
Non-physical job	478	60.1	453	57.0
Physical job	272	34.2	105	13.2
Inoccupation	45	5.7	237	29.8
Drinking				
Drinker	283	35.6	284	35.7
Non-drinker	512	64.4	511	64.3
Smoking				
Smoker	323	40.6	30	3.8
Non-smoker	472	59.4	765	96.2
Sleep duration				
≤6 h	342	43.0	351	44.2
7–8 h	419	52.7	422	53.1
≥9 h	34	4.3	22	2.7
Total	795	100.0	795	100.0

**Table 3:** Sleep duration according to the general characteristics of adolescents and parents.

Variable	Adolescent Sleep Duration			Total *n* (%)	χ^2^(*p*)
	≤6 h *n* (%)	7–8 h *n* (%)	≥9 h *n* (%)		
Adolescent					
Gender					
Male	158 (38.0)	207 (49.8)	51 (12.2)	416 (100.0)	8.897 (0.012 *)
Female	165 (43.5)	190 (50.1)	24 (6.4)	379 (100.0)	
House-hold Income					
High	130 (43.0)	155 (51.3)	17 (5.6)	302 (100.0)	9.305 (0.157)
Middle-high	100 (38.0)	132 (50.2)	31 (11.8)	263 (100.0)	
Middle-low	71 (39.2)	89 (49.2)	21 (11.6)	181 (100.0)	
Low	22 (44.9)	21 (42.9)	6 (12.2)	49 (100.0)	
Drinking					
Drinker	157 (45.2)	171 (49.3)	19 (5.5)	347 (100.0)	13.510 (0.001 **)
Non-drinker	66 (37.1)	226 (50.4)	56 (12.5)	448 (100.0)	
Smoking					
Smoker	19 (42.2)	21 (46.7)	5 (11.1)	45 (100.0)	0.274 (0.872)
Non-smoker	304 (40.5)	376 (50.1)	70 (9.3)	750 (100.0)	
Father					
Age					
≥50 yr	90 (45.9)	93 (47.4)	13 (6.6)	196 (100.0)	4.278 (0.118)
<50 yr	233 (38.9)	304 (50.8)	62 (10.4)	599 (100.0)	
Education level					
≥College	100 (39.4)	142 (55.9)	12 (4.7)	254 (100.0)	11.584 (0.003 **)
≤High school	223 (41.2)	255 (47.1)	63 (11.6)	599 (100.0)	
Occupation					
Non-physical job	199 (41.6)	240 (50.2)	39 (8.2)	478 (100.0)	4.512 (0.341)
Physical job	102 (37.5)	138 (50.7)	32 (11.8)	272 (100.0)	
Inoccupation	22 (78.9)	19 (42.2)	4 (8.9)	45 (100.0)	
Drinking					
Drinker	113 (39.9)	130 (45.9)	40 (14.1)	283 (100.0)	11.752 (0.003 **)
Non-drinker	210 (41.0)	267 (52.1)	35 (6.8)	512 (100.0)	
Smoking					
Smoker	127 (39.3)	157 (48.6)	39 (12.1)	323 (100.0)	4.443 (0.108)
Non-smoker	196 (41.5)	240 (50.8)	36 (7.6)	472 (100.0)	
Mother					
Age					
≥50 yr	43 (48.9)	36 (40.9)	9 (10.2)	88 (100.0)	3.333 (0.189)
<50 yr	280 (39.6)	361 (51.1)	66 (9.3)	707 (100.0)	
Education level					
≥College	87 (39.9)	118 (54.1)	13 (6.0)	218 (100.0)	4.930 (0.085)
≤High school	236 (40.9)	279 (48.4)	62 (10.7)	577 (100.0)	
Occupation					
Non-physical job	180 (39.7)	234 (51.7)	39 (8.6)	453 (100.0)	3.092 (0.542)
Physical job	45 (42.9)	52 (49.5)	8 (7.6)	105 (100.0)	
Inoccupation	98 (41.4)	111 (46.8)	28 (11.8)	237 (100.0)	
Drinking					
Drinker	109 (38.4)	130 (45.8)	45 (15.8)	284 (100.0)	21.333 (<0.001 ***)
Non-drinker	214 (41.9)	267 (52.3)	30 (5.9)	511 (100.0)	
Smoking					
Smoker	10 (33.3)	15 (50.0)	5 (16.7)	30 (100.0)	2.137 (0.343)
Non-smoker	313 (40.9)	382 (49.9)	70 (9.2)	765 (100.0)	
Total	323 (40.6)	397 (49.9)	5 (9.4)	795 (100.0)	

Note: * *p* < 0.05, ** *p* < 0.01, *** *p* < 0.001.

**Table 4:** Kappa statistics of the adolescents’ and parents’ sleep duration.

Variable	Adolescent Sleep Duration			Total *n* (%)	χ^2^(*p*) † Cohen Kappa (*p*)
	≤6 h *n* (%)	7–8 h *n* (%)	≥9 h *n* (%)		
Father sleep duration					
<6 h	159 (46.5)	161 (47.1)	22 (6.4)	342 (100.0)	12.156 (0.016 *)
7–8 h	151 (36.0)	218 (52.0)	50 (11.9)	419 (100.0)	† 0.064 (0.031 *)
≥9 h	13 (38.2)	18 (52.9)	3 (8.8)	34 (100.0)	Slight agreement
Mother sleep duration					
<6 h	190 (54.1)	135 (38.5)	26 (7.4)	351 (100.0)	53.925 (<0.001 ***)
7–8 h	124 (29.4)	254 (60.2)	44 (10.4)	422 (100.0)	† 0.213 (<0.001 ***)
≥9 h	9 (40.9)	8 (36.4)	5 (22.7)	22 (100.0)	Fair agreement
Total	323 (40.6)	397 (49.9)	75 (9.5)	795 (100.0)	

Note: † Cohen’s Kappa coefficient (Interpretation of Cohen’s Kappa statistic for strength of agreement. <0 = poor, 0.00–0.20 = slight agreement, 0.21–0.40 = fair agreement, 0.41–0.60 = moderate agreement, 0.61–0.80 = substantial agreement, 0.81–0.99 = near perfect agreement, 1 = perfect agreement), * *p* < 0.05, *** *p* < 0.001.

**Table 5:** Factors affecting 7–8 h sleep duration (ref: ≤6 h & ≥9 h).

Variable	B	OR	*p*	95% CI	
				Lower	Upper
Adolescent					
Gender Male (ref: Female)	−0.058	0.943	0.697	0.703	1.266
Household income Middle-low (ref: Low)	−0.022	0.978	0.950	0.489	1.956
Household income Middle-high (ref: Low)	−0.019	0.981	0.956	0.494	1.948
Household income High (ref: Low)	0.058	1.060	0.869	0.529	2.124
Drinking Non-drinker (ref: Drinker)	0.241	1.273	0.135	0.928	1.747
Smoking Non-smoker (ref: Smoker)	0.160	1.173	0.627	0.617	2.231
Father					
Age ≤50 yr (ref: ≥50 yr)	−0.178	0.837	0.383	0.562	1.248
Education level ≥College (ref: High school)	0.317	1.373	0.153	0.889	2.122
Occupation Non-physical job (ref: Inoccupation)	0.140	1.150	0.697	0.569	2.327
Occupation Physical job (ref: Inoccupation)	0.204	1.226	0.566	0.612	2.458
Drinking Drinker (ref: Non-drinker)	−0.023	0.977	0.925	0.601	1.588
Smoking Smoker (ref: Non-Smoker)	−0.175	0.839	0.257	0.620	1.136
Sleep duration 7–8 h (ref: ≤6 h & ≥9 h)	0.098	1.103	0.512	0.822	1.481
Mother					
Age ≤50 yr (ref: ≥50 yr)	−0.276	0.759	0.310	0.445	1.293
Education level ≥College (ref: High school)	−0.042	0.959	0.845	0.628	1.464
Occupation Non-physical job (ref: Inoccupation)	0.206	1.229	0.230	0.878	1.720
Occupation Physical job (ref: Inoccupation)	0.269	1.309	0.289	0.796	2.152
Drinking Drinker (ref: Non-drinker)	−0.327	0.721	0.148	0.464	1.123
Smoking Smoker (ref: Non-Smoker)	0.200	1.222	0.609	0.567	2.632
Sleep duration 7–8 h (ref: ≤6 h & ≥9 h)	0.914	2.494	<0.001 ***	1.850	3.362
Constant	−0.941	0.390	0.083		

Note: OR = odds ratio, CI = confidence interval, *** *p* < 0.001.

## Data Availability

KNHANES data can be accessed and downloaded from the KNHANES website (https://knhanes.cdc.go.kr/knhanes/index.do accessed on 20 May 2021).
